# A Case of Pediatric Mixed Epithelial and Stromal Tumor of the Kidney With Atypical Stromal Cells

**DOI:** 10.7759/cureus.71661

**Published:** 2024-10-16

**Authors:** Takahiro Tsuchiya, Keiichiro Uehara, Tomonori Tanaka, Yasuyoshi Okamura, Chisato Ohe, Kenichi Kohashi, Tomoo Itoh

**Affiliations:** 1 Department of Diagnostic Pathology, Kobe University Hospital, Kobe, JPN; 2 Department of Pathology, Yodogawa Christian Hospital, Osaka, JPN; 3 Department of Urology, Kobe University Hospital, Kobe, JPN; 4 Department of Pathology, Graduate School of Medicine, Osaka Metropolitan University, Osaka, JPN

**Keywords:** atypical stromal cells, kidney, mestk, mixed epithelial and stromal tumor of the kidney, pediatric

## Abstract

Mixed epithelial and stromal tumor of the kidney (MESTK) is a rare renal tumor characterized by both cystic and solid components. Although typically benign, its components can undergo malignant transformation, manifesting as a sarcomatous feature. Carcinomatous transformations are exceedingly rare. MESTK predominantly affects perimenopausal women, with male patients being rare and often associated with a history of hormonal therapy. Pediatric MESTK is extremely rare, with few reports. We herein report an eight-year-old child diagnosed with MESTK, exhibiting typical histological findings and featuring a few stromal cells with atypia in a focal area. These atypical cells exhibited bizarre nuclei and were positive for p53, although no mitotic figures were observed, and the Ki-67 labeling index was not elevated compared with the surrounding areas. The follow-up period was relatively short and there was no evidence of recurrence or metastasis. The patient remains under careful observation.

## Introduction

Mixed epithelial and stromal tumor of the kidney (MESTK) is a distinctive biphasic renal cell tumor characterized by epithelial cysts lined by benign renal epithelium and proliferation of cytologically benign, spindle-shaped stromal cells [1]. MESTKs are almost exclusively diagnosed in middle-aged women, and their occurrence in men is extremely rare, with some cases associated with a history of hormone therapy [2]. Its incidence in pediatric populations is exceedingly rare, with only six reported cases, highlighting the exceptional nature of this condition in children [3-7].

Although MESTKs generally exhibit a favorable prognosis and are considered benign, there have been documented instances of malignant transformation in adult patients. Almost all transformed MESTKs presented a sarcomatous phenotype. The clinical outcomes of these cases vary, ranging from a short-term prognosis despite postoperative treatment to a long-term survival without recurrence or metastasis [8].

In this context, we present a pediatric case of MESTK, characterized by the presence of atypical stromal cells, raising concerns regarding malignancy. This case contributes to the limited literature on pediatric MESTK and underscores the importance of a careful histopathological assessment of atypical presentations.

## Case presentation

**Figure 1:**
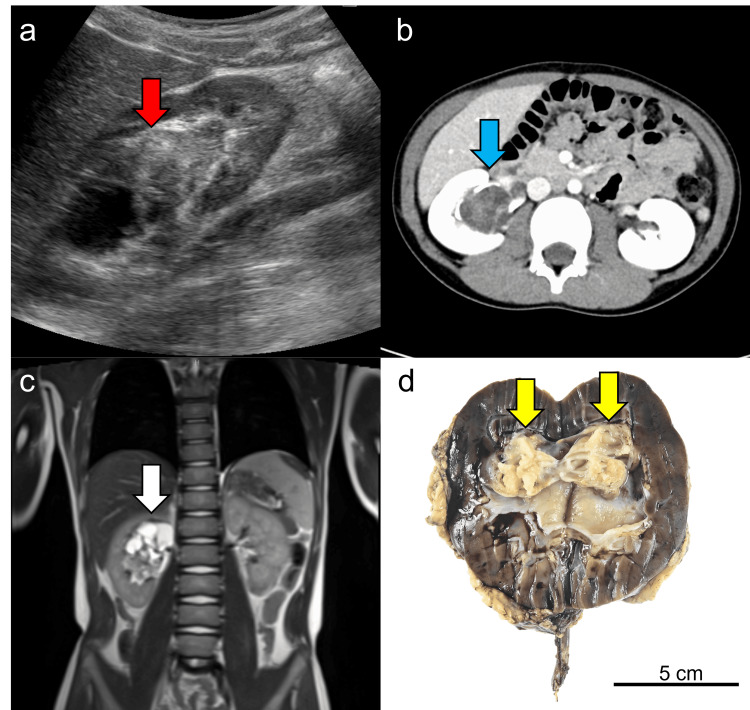
Imaging and gross examination (a) Ultrasonography shows an oligemic mass with cystic lesion (red arrow). (b) CT shows a well-demarcated 4 × 4 × 3 cm mass at the hilum of the right kidney (blue arrow). (c) T2-weighted MRI depicts a tumor situated at the upper pole of the right kidney, characterized by a cystic appearance (white arrow). (d) The total nephrectomy specimen shows a mixed cystic and solid tumor with a white-tan appearance protruding into the renal pelvis in the resected kidney (yellow arrow).

An eight-year-old boy presented with intermittent albuminuria, which has been prevalent since he was six years old. Aside from albuminuria, he had no significant personal or family history of disease. Ultrasonography revealed an oligemic mass measuring 3.1 cm at the upper pole of the right renal pelvis (Figure 1a). Computed tomography (CT) further delineated a well-demarcated 4 × 4 × 3 cm mass at the hilum of the right kidney, characterized by multiple cystic components and mild contrast enhancement (Figure 1b). Magnetic resonance imaging (MRI) showed low-signal lesions in the cystic area on T1-weighted images and high-signal lesions on T2-weighted images, indicating that the contents were predominantly water. Both T1- and T2-weighted images showed equal signals in the interstitial area (Figure 1c). Given that a needle biopsy of the tumor did not exclude malignancy, radical nephrectomy was performed. A preoperative PET-CT scan was conducted to evaluate for metastatic disease; however, no metastatic lesions were identified. A gross examination of the resected kidney revealed a white tumor measuring 3 cm, containing both cystic and solid components, located at the upper pole of the kidney and protruding into the renal pelvis (Figure 1d).

**Figure 2:**
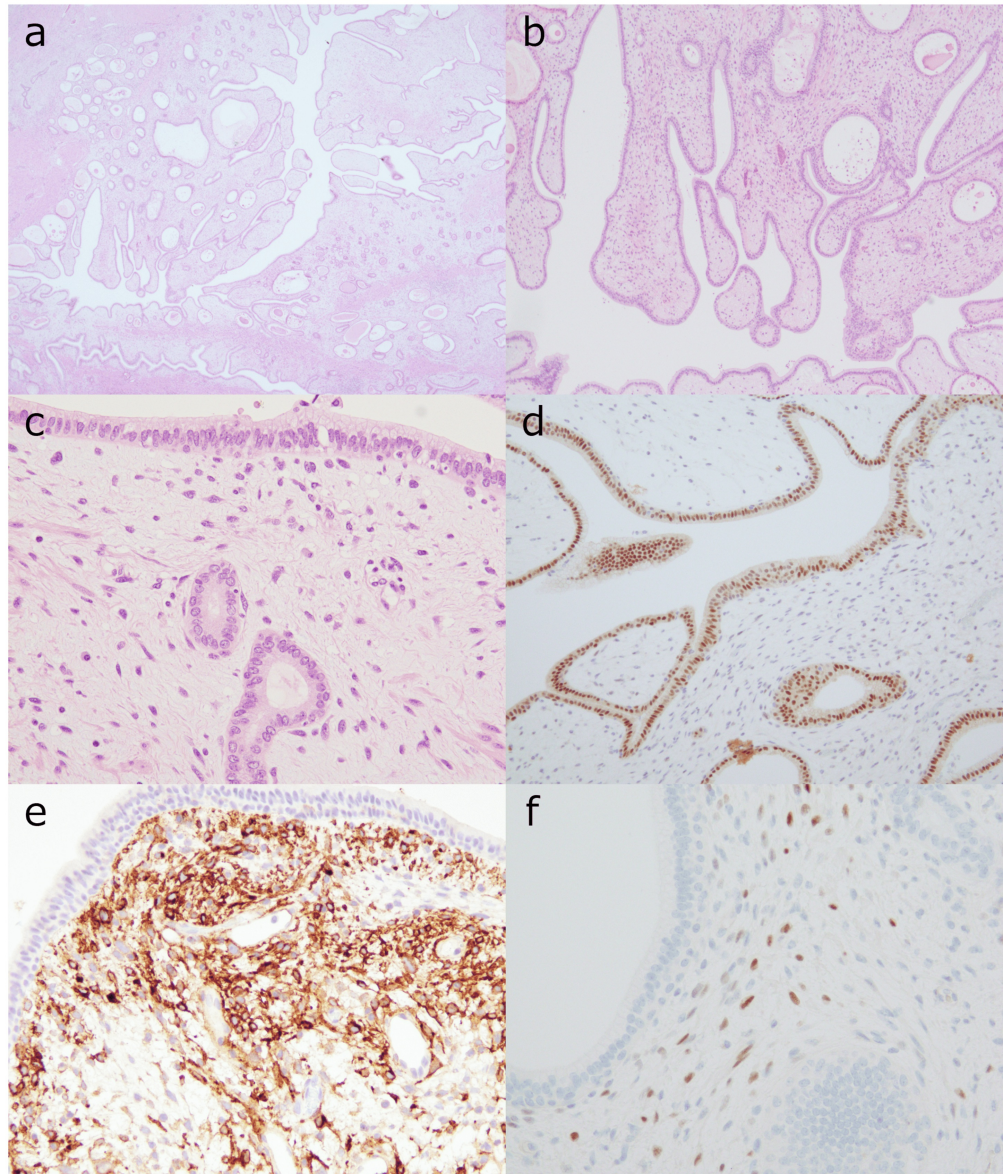
Histopathological findings in a typical region of the MESTK (a, b) Variable-sized cysts and glands are interspersed within fibrous, edematous, or myxoid stroma. A phyllodes-like structure was observed in the low-power field. (c) The epithelial cells exhibit a cuboidal or columnar morphology, while subepithelial stromal cells are non-specific or spindle-shaped. Both epithelial and stromal cells in most areas display no atypia. Immunohistochemically, the epithelial cells are positive for Pax8 (d). The stromal cells are diffusely positive for CD10 (e) and show partial positivity for estrogen receptor (ER) (f). MESTK: mixed epithelial and stromal tumor of the kidney

A histopathological evaluation indicated that the tumor contained both epithelial and stromal components. Variable-sized glands and cysts were scattered throughout the tumor and were lined by epithelial cells, which appeared predominantly flat and cuboidal to columnar (Figures 2a, 2b). Some areas showed a phyllodes-like appearance, similar to that observed in the breast fibroepithelial tumors (Figure 2a). In some areas, epithelial cells exhibited stratification resembling that of the urothelium. The glands and cysts were surrounded by fibrous stroma with focal myxoid or edematous changes, composed of non-specific spindle cells (Figure 2c). Immature or ectopic elements, such as skeletal muscle and cartilage, were not observed. Immunohistochemical staining (IHC) showed that epithelial cells of the glands and cysts within the tumor were diffusely positive for Pax8 (Figure 2d). The stromal spindle cells were positive for CD10 and estrogen receptor (ER) in some areas (Figures 2e, 2f). No inhibin- and calretinin-positive luteinized stromal spindle cells were detected in the tumor.

**Figure 3:**
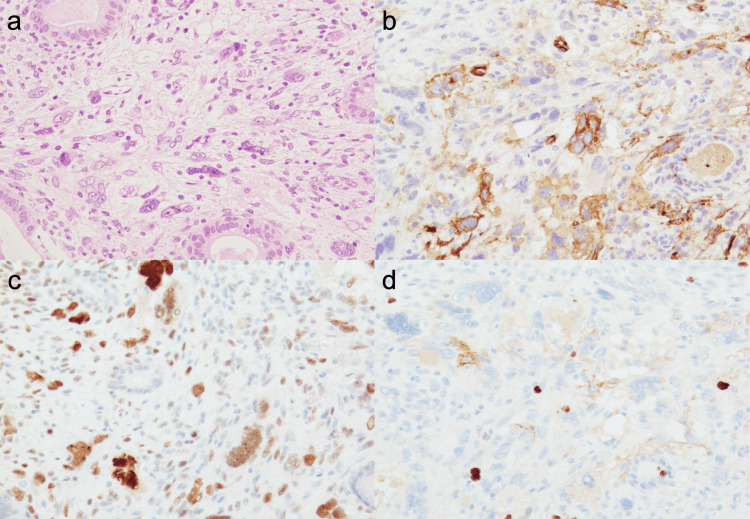
Immunohistochemical findings of atypical stromal cells in the MESTK (a) Atypical stromal cells with bizarre nuclei focally seen within the tumor. Neither mitotic figures nor necrosis are present. These atypical cells are positive for CD10 (b) and p53 (c). The atypical cells are mostly negative for Ki-67 (d), and the Ki-67 labeling index is not significantly higher than the surrounding area. MESTK: mixed epithelial and stromal tumor of the kidney

These immunohistological findings were consistent with a diagnosis of MESTK. However, pleomorphic stromal cells were observed in the focal area of the tumor (Figure 3a). These cells were positive for CD10 (Figure 3b) and negative for ER, SSX18-SSX, and SSX. Mitotic figures were not observed among the atypical cells. IHC for p53 showed a wild pattern of positivity in most areas of the tumor; however, some atypical stromal cells were strongly positive (Figure 3c). The Ki-67 labeling index among these pleomorphic cells was not much higher than that in the surrounding area, and almost all atypical stromal cells were negative (Figure 3d). There were no mitotic figures, necrosis, invasion of the renal parenchyma, lymphovascular invasion, or intrarenal metastases. Although the patient’s age was atypical, MESTK was diagnosed based on hematoxylin and eosin (H&E) morphology and IHC.

No postoperative adverse events were observed. During the six-month follow-up period after surgery, no disease recurrence or progression was observed.

## Discussion

MESTK is a rare renal tumor comprising a mixture of cystic and solid areas. The gross appearance of this tumor typically shows a well-circumscribed, unencapsulated mass with no infiltrative growth into the renal parenchyma, pelvicalyceal system, or renal vessels, although it may appear to replace these sites with compression [2]. The mass includes cystic and solid components; the solid lesions are gray to white, while cystic lesions vary in size [1]. Histologically, variable-sized cysts and glands are separated by stroma. Some glands and cysts have complex, phyllode-like structures. Epithelial cells lining cysts or glands are typically flat and cuboidal with variability in cell types, including hobnail-shaped, urothelial, ciliated, clear cells, mucinous goblet cells, and endometrioid differentiation. Septal stroma usually shows fibrous tissue, sometimes with edematous or myxoid changes. Immunohistochemically, epithelial cells are positive for Pax2 or Pax8, whereas the stromal component is typically positive for actin, desmin, CD10, ER, and PgR. Luteinized stromal cells are positive for inhibin and calretinin [1]. Both epithelial and stromal cells are considered clonal in origin [9].

The origin of this tumor is hypothetical; one theory suggests that it is derived from the primary mesenchyme/metanephric blastema and another from the periductal fetal mesenchyme, which may have the capacity to interact with the epithelia [2]. MESTK primarily affects middle-aged women, and most patients are asymptomatic, although symptoms such as hematuria and abdominal pain can occur [1]. Rare male cases have been linked to a history of hormone therapy, specifically estrogen therapy for prostate cancer. This situation suggests that the periductal fetal mesenchyme around the epithelial component in the renal medulla may proliferate under female hormone stimulation [2]. Congenital mesoblastic nephroma (CMN) is a differential disease for MESTK, but it can be ruled out based on age, as CMN develops congenitally or by age three with 90% of cases occurring in the first year of life [1]. Additionally, the epithelial component of CMN usually involves preexisting urothelium and is located at the periphery of the lesion, whereas the epithelial component in MESTK, like in this case, is found throughout the lesion, forming phyllodes tumor-like structures that indicate the proliferation of both epithelial and stromal components. Cystic partially differentiated nephroblastoma is another differential diagnosis, as it occurs in pediatric kidneys [5]. However, such a tumor typically contains immature or ectopic elements, such as skeletal muscle or cartilage, which were not observed in this case. Synovial sarcoma also needs to be considered, as it involves the proliferation of stromal spindle-shaped cells. However, immunostaining for SSX and the SS18-SSX fusion gene, typically positive in synovial sarcoma, was negative in this case, allowing us to rule out this diagnosis [10].

To our knowledge, six pediatric cases of MESTK before puberty have been reported (Table 1) [3-7]. The cases reported by Wei et al. were not typical MESTK in morphology, exhibiting an almost entirely solid appearance, as described in their article [7]. However, the stromal spindle cells were positive for ER immunostaining, leading to the classification of these tumors as pediatric MESTK. Four of these cases were found in the second decade of life, whereas the other three, including our case, were in the first decade. Unlike adult cases, male cases are more predominant than female cases in pediatric MESTK. Three cases were discovered due to hematuria, and two cases presented with an abdominal mass. A presentation of albuminuria, as in our case, has not been previously reported. Reported pediatric MESTKs have ranged in a maximum diameter from 2.0-16.9 cm, averaging 8.2 cm. Histologically, our case was typical in terms of both histological and immunostaining findings, except for atypical stromal cells.

**Table 1:** Reported pediatric MESTK cases MESTK: mixed epithelial and stromal tumor of the kidney

Cases	Age (years)	Gender	Clinical symptoms	Location	Max diameter	Treatment	Follow-up duration	Recurrence
Hara et al., 2005 [3]	12	Female	Abdominal mass	Right kidney lower pole	14.0 cm	Radical nephrectomy	40 months	No recurrence
Choy et al., 2012 [4]	14	Male	Microscopic hematuria	Right kidney lower pole	2.0 cm	Partial nephrectomy	9 months	No recurrence
Teklali et al., 2010 [5]	12	Male	Hematuria	Left kidney upper pole	4.5 cm	Partial nephrectomy	48 months	No recurrence
Gibson et al., 2019 [6]	14	Male	Blunt trauma Hematuria	Right kidney anteromedial	7.0 cm	Radical nephrectomy	18 months	No recurrence
Wei et al., 2023 [7]	3	Female	Hematuria	Left kidney	10.2 cm	Radical nephrectomy	3-6 months	No recurrence
Wei et al., 2023 [7]	4	Male	Abdominal mass	Left kidney	16.9 cm	Partial nephrectomy	3-6 months	No recurrence
Our case	8	Male	Albuminuria	Right kidney upper pole	3.0 cm	Radical nephrectomy	6 months	No recurrence

Such tumors with typical features are benign [1]. However, malignant transformation of MESTK in adults has been reported in approximately 30 cases [8,11,12]. While cases in women predominate, a few in men have also been reported. Most reported cases involved stromal malignant transformation and unclassified sarcomas, followed by synovial sarcomas and rhabdomyosarcoma [8]. One case of Ewing sarcoma in MESTK has been previously reported [11]. Some cases exhibit epithelial malignant transformation, such as adenocarcinomas, or both, such as carcinosarcomas. In our case, the epithelial cells showed no atypia, whereas atypical stromal cells with enlarged and bizarre nuclei were positive for p53 in the focal area of the tumor. However, there was no apparent evidence of sarcomatous transformation, as mitotic figures, necrosis, and increased cell density were not observed. Pediatric MESTK with malignant transformation has not been described; all six previous cases lacked atypical cells, and recurrence of pediatric MESTK was not reported during the follow-up periods of 3-40 months [3-7].

## Conclusions

Pediatric MESTK is extremely rare, and no cases of recurrence or malignant transformation have been reported to date. To our knowledge, this is the first reported case of pediatric MESTK with atypical stromal cells. The follow-up period for this case after surgery was only six months, which is a short duration to definitively determine the benign or malignant nature of the tumor. The patient survived without recurrence or metastasis to other organs. As the tumor was completely resected, we planned to observe the patient carefully, primarily through radiological examinations. 

The presence of atypical stromal cells in pediatric MESTK poses a diagnostic challenge. While the clinical behavior of this tumor is generally benign, the significance of these atypical cells remains unclear. Further accumulation of cases and longer follow-up periods are necessary to better understand the role of atypical stromal cells in pediatric MESTK.
